# Effect of Psychological Inflexibility on Depressive Symptoms and Sleep Disturbance among Japanese Young Women with Chronic Pain

**DOI:** 10.3390/ijerph17207426

**Published:** 2020-10-12

**Authors:** Tsukasa Kato

**Affiliations:** Department of Social Psychology, Toyo University, Tokyo 112-8606, Japan; mtsukasa@hotmail.com

**Keywords:** acceptance and commitment therapy, Asian, depression, female, insomnia, pain intensity, psychological flexibility, sleep disorder

## Abstract

Background: Psychological inflexibility based on an acceptance and commitment therapy model is theoretically well-established as a process to exacerbate psychological distress, such as depressive symptoms and sleep disturbance. This study aimed to examine the associations of psychological inflexibility with depressive symptoms and sleep distribution. We hypothesized that psychological inflexibility would be associated with higher levels of depressive symptoms and sleep disturbance in women with chronic pain. Methods: Female college students in Japan answered a questionnaire on pain status, treatment, and psychological inflexibility as measured by the Acceptance and Action Questionnaire-Ⅱ before answering questionnaires on depressive symptoms and sleep disturbance eight months after. Results: Women with chronic pain (*n* = 320) reported more severe depressive symptoms and sleep disturbances compared to women without chronic pain (*n* = 90). Hierarchical multiple regressions revealed that psychological inflexibility predicted higher levels of depressive symptoms and sleep disturbance, independent of the pain intensity, whether they consulted a doctor or used pain medication. Conclusion: Based on our findings self-management interventions aimed at reducing psychological inflexibility should be developed for individuals who are experiencing chronic pain.

## 1. Introduction

Chronic pain is defined by a specific period of time that pain persists; although the period is arbitrarily defined each study, it commonly lasts for three or six months [1]. Chronic pain has a high prevalence rate [1]; the prevalence rate in women is higher than that in men [2]. Women also differ from men in their responses to pain, such as biological responses, emotions, and strategies for managing pain, and they have increased pain sensitivity [3,4]. Therefore, it is useful to focus on women with chronic pain.

Depressive symptoms and sleep disturbance are common in patients with chronic pain [5,6], and depression, sleep disturbance, and chronic pain share abnormal patterns of brain activation, such as increased limbic activity [5]. Specifically, approximately 35% of individuals diagnosed with chronic pain have comorbid depression [7], and the association between chronic pain and depressive symptoms became stronger as the severity and frequency of pain increased [8,9,10]. Between 50 and 90% of individuals with chronic pain are diagnosed with complaints of sleep disturbance [5]; pain intensity and frequency reported by individuals with chronic pain were associated with the severity of sleep disturbance [11,12,13]. Additionally, decreased brain-derived neurotrophic factor and 5-hydroxytryptamine have been observed among patients with depression, sleep disturbance, and chronic pain [5].

### 1.1. Psychological Inflexibility for Chronic Pain

Psychological inflexibility further exacerbates depressive symptoms and sleep disturbance in individuals with chronic pain. Psychological inflexibility is a core concept in the acceptance and commitment therapy (ACT) model [14], which is rooted in functional contextualism and contextual behavioral science. Psychological inflexibility is defined as one’s inability to fully focus on the present moment, according to what the situation demands, and the inability to change or persist with behavior to pursue goals and values [14]. In addition, it includes six subprocesses (i.e., avoidance, fusion, preoccupation with past or future, self-as-content, values-inconsistent action, and inaction impulsive persistence). Psychological inflexibility can explain the process by which aversive private experiences (i.e., thoughts, feelings, and sensations) and adverse outcomes produce behavioral patterns that contradict an individual’s values and goals. For example, some individuals engage in maladaptive avoidance behaviors, such as binge eating, binge drinking, and pain-related injustice, to avoid aversive situations and uncomfortable emotions when attempting to control or eliminate negative experiences. Such avoidance behaviors lead to more negative emotions, which, in turn, lead individuals to engage in more maladaptive avoidance behaviors. Consequently, such avoidance produces chronic symptoms of distress [14].

A psychological inflexibility model for chronic pain [15,16] predicts that the process of psychological inflexibility leads to the experience of pain-related injustice to problematic chronic pain outcomes. The experience of pain-related injustice reflects one’s appraisal regarding pain-related loss, externalized blame, and unfairness, and it induces aversive experiences for some individuals. Circumstances may be perceived as unfair and may produce emotional distress. Repeated failed attempts to solve the problem of pain-related injustice may lead individuals to further experience injustice and engage in escape activities that are important to them. Consequently, the experience of pain-related injustice generates chronic symptoms of distress in individuals with chronic pain.

In fact, accumulating evidence suggests that psychological inflexibility is associated with higher levels of depressive symptoms in individuals with chronic pain [17,18,19,20,21,22,23,24]. Additionally, ACT for chronic pain alleviates depressive symptoms by improving psychological inflexibility [22,25,26,27,28,29,30]. One study [31] found that psychological inflexibility predicted severe depressive symptoms in 144 patients with chronic pain, independent of the effect of their pain intensity, mindfulness, and pain-related acceptance. The finding that psychological inflexibility plays a unique role in depressive symptoms beyond the effect of pain intensity has been replicated [17,18,19,20,21,22,23,24]. Moreover, another study [20] on patients with chronic pain conducted an ACT-based treatment for chronic pain and found that psychological inflexibility, pain intensity, and depressive symptoms at the three-month follow-up was reduced by the treatment, and that the change in score of psychological inflexibility at pre-treatment and follow-up was associated with that of depressive symptoms even when controlling for the effects of pain intensity and mindfulness.

Regarding sleep disturbance, psychological inflexibility is associated with more severe sleep disturbance in individuals with chronic pain [26,32]. For example, one study [33] of patients attending a pain management center found that psychological inflexibility was associated with higher levels of insomnia and problems with sleep and rest. However, psychological inflexibility was not associated with either insomnia severity nor problems with sleep and rest, when controlling for the effects of pain intensity and pain duration. Another study of patients with chronic pain found that pain intensity, pain interference, insomnia severity, and sleep interference at nine months post-treatment were reduced by a treatment for chronic pain based on ACT, and that the change in score of psychological inflexibility between pre-treatment and follow-up was associated with those of insomnia severity and sleep interference, independent of the effects of pain intensity and pain interference. However, studies on individuals with chronic pain that examined the association between psychological inflexibility and sleep are very scant in relation to the association between psychological inflexibility and depressive symptoms.

### 1.2. Psychological Inflexibility for Chronic Pain in Asia

Although the process of psychological inflexibility has been applied mostly in Western cultures, its evidence remains limited in Asia [24]. This tendency is particularly evident in research on chronic pain. In fact, when entering “psychological inflexibility” and “chronic pain” and “a country’s name” (i.e., one of 52 Asian countries including China, Japan, and India), as an “all field” into the PubMed database, only five studies [18,24,34,35,36] came up. Three out of the five studies examined the association of psychological inflexibility with depressive symptoms, and these three showed similar findings across Western cultures. For example, Kato et al. [18], who used a cross-sectional design, found that psychological inflexibility predicted higher levels of depressive symptoms in both samples of Japanese individuals with chronic pain and menstrual pain, even when controlling for the effects of pain intensity and pain acceptance. However, none of these studies explored the association between psychological inflexibility and sleep disturbance.

In this study, the associations of psychological inflexibility with depressive symptoms and sleep disturbance were examined in women with chronic pain using a longitudinal design. Controlling for the effect of pain intensity, we hypothesized that psychological inflexibility would be associated with higher levels of depressive symptoms and sleep disturbance among women with chronic pain, all of which were measured eight months after the beginning of the study.

## 2. Materials and Methods

### 2.1. Participants and Procedure

After providing written informed consent, the 750 potential participants were asked to answer several questions on their physical health status, lifestyle, and biological sex. Additionally, they were asked to describe the most severe pain (e.g., pain site, pain status, and pain intensity) that they had experienced during the last year and state the duration of the pain that they had described. This was the same procedure used in the previous study [18]. For this study, 431 women were selected from the 750 potential participants. The exclusion criteria for this study were women who had not experienced pain during the last year, had not experienced hospitalization or outpatient for any mental disorders, smoked tobacco cigarettes, consumed alcoholic beverages excessively, and all men. They completed a questionnaire that measured psychological inflexibility (Wave 1), and they completed each questionnaire that measured depressive symptoms and sleep disturbance (Wave 2) approximately eight months after Wave 1. Twenty-one women dropped out. Therefore, as a result, 410 women ranging from 18 to 27 years (*M* = 18.64, SD = 0.84) participated in this study. These data were not influenced by COVID-19 because the data had been collected by February 2020.

In this study, the participants were classified depending on the presence or absence of chronic pain and also by pain duration: more than three months. This criterion has been frequently used in pain research and clinical diagnoses [2], such as the 11th revision of the International Classification of Diseases (ICD-11) and the International Association for the Study of Pain.

All procedures followed were in accordance with the ethical standards of the responsible committee on human experimentation and with the Helsinki Declaration of 1975, as revised in 2000.

### 2.2. Measures

All instructions and scale items were written in Japanese for this study. Participants were instructed to answer in the context of the pain that they had described.

#### 2.2.1. Pain Status and Treatment

Pain intensity was measured using the 11-point pain intensity numerical rating scale (11-point NRS). The reliability and validity of the 11-point NRS have already been established in previous studies [37,38]. Participants rated the average pain intensity of the pain described as the most severe over the last week. According to the ICD-11 [39], the pain intensity was categorized into three groups (low 0–3, moderate 4–6, and severe 7–10); each category was coded (low = 1, moderate = 2, and severe = 3) for multiple regression analyses. In this study, no participant in the chronic pain sample reported a zero score for pain intensity, and only one participant reported a score of one for pain intensity.

Moreover, participants were asked about medical treatment in the hospital (i.e., inpatient treatment, regular outpatient treatment, formal medical advice, or no medical treatment) and their medication use (i.e., prescription medication, nonprescription medication, or no medication). Medical treatment in the hospital and medication use were coded as 0 (No) and 1 (Yes), respectively, for multiple regression analyses.

#### 2.2.2. Psychological Inflexibility for Pain

Psychological inflexibility for pain was assessed using the Japanese version [40] of the Acceptance and Action Questionnaire-Ⅱ (AAQ-II) [41]. The AAQ-II has been applied across diverse samples [42], including patients and individuals with chronic pain [19,20,21,22,23,24]. Additionally, the reliability and validity of the AAQ-II for chronic pain have already been established in previous studies [31]. The Japanese version was positively associated with higher depressive symptoms and sleep difficulty [40]. Additionally, the AAQ-II score of the Japanese version predicted depressive symptoms in people with chronic pain, even after controlling for the effect of pain acceptance as measured by the Chronic Pain Acceptance Questionnaire-8 [18]. The Japanese version has also been utilized for individuals with chronic pain, with a Cronbach’s alpha of 0.89 for the sample with chronic pain and 0.91 for the sample with menstrual pain [18]. In this study, participants responded each item on a seven-point Likert scale (0 = never true to 6 = always true). A higher AAQ-II score indicates higher levels of psychological inflexibility. In this study, Cronbach’s alpha for the AAQ-II was 0.90.

#### 2.2.3. Depressive Symptoms

Depressive symptoms were measured using the Japanese version [40] of the Patient Health Questionnaire-9 (PHQ-9) [43]. The PHQ-9 has been used to treat depressive symptoms in individuals and patients with chronic pain [17,22,24,26,30]. The Japanese version is strongly correlated with another scale designed to measure depressive symptoms [44]. Additionally, the Cronbach’s alphas for the Japanese version were 0.83 for a relatively large sample of college students (*n* = 1468) [44] and 0.82 for the sample with chronic pain [18]. In this study, participants rated the degree to which they had experienced each item during the past two weeks on a four-point Likert scale (0 = *not at all* to 3 = *nearly every day*). A higher PHQ-9 score suggests higher depressive symptoms. The Cronbach’s alpha for the PHQ-9 was 0.86 in this study.

#### 2.2.4. Sleep Disturbance

Sleep disturbance was measured using the Japanese version [45] of the Epworth Sleepiness Scale (ESS) [46], which was designed to measure excessive daytime sleepiness. The reliability and validity of the original ESS are well-established in multiple cultures and ethnicities and across diverse samples, such as individuals with chronic pain [47]. The Japanese version of the ESS was correlated with other scales of sleep disturbance, sleep duration, and sleep latency, and its Cronbach’s alphas ranged from 0.71 to 0.73 [45]. Participants rated the chances of dozing off or falling asleep while they were engaged in different activities (eight items) within the past two weeks on a four-point Likert scale ranging from 0 (*would never doze or sleeping*) to 3 (*high change of dozing or sleep*). A higher ESS score indicates higher levels of sleep disturbance. The Cronbach’s alpha for the PHQ-9 was 0.71 in this study.

### 2.3. Data Analysis

Hierarchical multiple regression analyses for women with chronic pain were conducted using depressive symptoms or sleep disturbance measured at Wave 2 as a dependent variable. Medical treatment in the hospital (0 = No and 1 = Yes), medication use (0 = No and 1 = Yes), and pain intensity scores were entered into the first step of the regression equation. Afterwards, a psychological inflexibility score was entered into the second step of the regression equation. Additionally, an interaction score between pain intensity and psychological inflexibility was entered into the third step to examine whether psychological inflexibility moderated the relationship between pain intensity and depressive symptoms or sleep disturbance, although this assumption was not hypothesized in this study. In this analysis, a prior power analysis was conducted with a medium effect size (*f*^2^ = 0.15), an alpha error probability of 0.05, and a statistical power of 0.90. The results showed that adequate sample sizes were 73 for delta *R*^2^ and 59 for a single regression coefficient, indicating that our sample size of 320 was sufficient for this analysis.

## 3. Results

A total of 78.0% (*n* = 320) of the 410 participants had a pain duration of more than three months (Table 1). Table 2 shows the means and standard deviations of each sample in the women with and without chronic pain. Additionally, to examine the representativeness of our sample, Table 2 shows the means and standard deviations of previous study (*n* = 473) [18], which were collected from some female college students in Japan using a considerably similar procedure (i.e., the same questions and coding) to that of our study. Both the depressive symptoms and sleep disturbance scores of women with chronic pain were higher than those of women without chronic pain. However, both depressive symptoms and psychological inflexibility scores of women with chronic pain were similar to those of the comparative sample. Specifically, although the depressive symptoms score in our sample was slightly higher than that of the comparative sample, the difference was not significant.

The psychological inflexibility scores were significantly and positively correlated with both depressive symptoms (*r* = 0.57, *p* < 0.001) and sleepiness scores (*r* = 0.17, *p* = 0.002) in our sample of women with chronic pain. A hierarchical multiple regression analysis for women with chronic pain using depressive symptoms scores revealed a significant delta *R*^2^: Δ*R*^2^ = 0.30, Δ*F* (1, 315) = 145.98, *p* < 0.001, effect size Cohen’s *f*^2^ = 0.23 (Table 3). Both *R*^2^s at Steps 1 and 2 were significant; *R*^2^ = 0.04, *F* (3, 316) = 4.71, *p* = 0.003, and Cohen’s *f*^2^ = 0.04 for Step 1; *R*^2^ = 0.35, *F* (4, 315) = 41.65, *p* < 0.001, and Cohen’s *f*^2^ = 0.25 for Step 2. However, no significant interaction score between pain intensity and psychological inflexibility was found at Step 3, Δ*R*^2^ = 0.01, Δ*F* (1, 314) = 1.57, *p* = 0.212, effect size Cohen’s *f*^2^ = 0.01.

A hierarchical multiple regression analysis for women with chronic pain using sleep disturbance scores showed a significant delta *R*^2^: Δ*R*^2^ = 0.03, Δ*F* (1315) = 9.91, *p* = 0.002, effect size Cohen’s *f*^2^ = 0.03 (Table 4). *R*^2^ at Step 1 was non-significant, *R*^2^ = 0.01, *F* (3316) = 1.04, *p* = 0.374, and Cohen’s *f*^2^ = 0.01, whereas *R*^2^ at Step 2 was significant, *R*^2^ = 0.04, *F* (4315) = 3.28, *p* < 0.012, and Cohen’s *f*^2^ = 0.04. However, no significant interaction between pain intensity and psychological inflexibility was found at Step 3, Δ*R*^2^ = 0.01, Δ*F* (1314) = 1.72, *p* = 0.190, effect size Cohen’s *f*^2^ = 0.01.

## 4. Discussion

Both multiple regression analyses on depressive symptoms and sleep disturbance showed significant Δ*R*^2^s when entering psychological inflexibility into each regression equation in Step 2. Psychological inflexibility did not moderate the relationship between pain intensity and depressive symptoms or sleep disturbance. Thus, higher levels of psychological inflexibility were associated with higher levels of depressive symptoms and sleep disturbance, independent of the effects of pain intensity, medical treatment, and medical use. These findings indicate that our hypothesis was supported in the Japanese sample of female college students with chronic pain. This finding in the Asian sample is consistent with that in Western samples, confirming the effect of psychological inflexibility in an Asian culture. It should be noted that some studies [48,49] found that psychological inflexibility as measured by the AAQ-Ⅱ among Asian Americans was higher than among White and Black Americans, and the association between psychological inflexibility and depressive symptoms in Asian Americans was weaker than that in White and Black American college students, who did not have chronic pain.

The representativeness of our sample was partially ensured by the comparisons between women with chronic pain in our sample and those in the comparative sample. Moreover, the results that depressive symptoms and sleep disturbance in women with chronic pain were more severe than those in women without such pain were consistent with previous studies [5,6]. These results suggest the overall validity of our data.

In addition to psychological inflexibility, individual traits such as alexithymia (difficulty to identify and distinguish feelings), rumination (repetitive thoughts focusing individuals’ attention on their depression), and neuroticism, are related to higher levels of depressive symptoms and sleep disturbance in individuals with chronic pain. Therefore, further research on the associations between these traits and psychological inflexibility may contribute to better understanding the role of psychological inflexibility in chronic pain.

Individuals with chronic pain can reduce pain-induced negative emotions and problems, such as depressive symptoms and sleep disturbance, by improving psychological inflexibility using intervention for chronic pain based on the ACT model, of which the efficacy is well-established in Western cultures [15,16]. Our findings may help individuals with chronic pain improve psychological inflexibility by means of ACT. Moreover, recently, some self-management practices for chronic pain based on the ACT model have been developed, and their efficacy has been reported [50,51]. Such self-management of chronic pain has received much attention. For example, clinical guidelines for chronic pain [52,53] emphasize the importance of self-management in attenuating pain-induced negative emotions and problems. Nevertheless, many individuals with chronic pain have used undesirable self-management, specifically self-medication with over-the-counter (OTC) analgesics to relieve pain [54], which may lead to possible adverse effects if used inappropriately [52]. In our sample, 36.6% of women with chronic pain used OTC analgesics. Interestingly, although the use of medication reduced depressive symptoms when controlling for the effects of pain intensity, its effect disappeared after controlling for the effect of psychological inflexibility. A similar finding emerged in previous studies [17,21,31]. This finding suggests that, for some women with chronic pain, the improvement of psychological inflexibility without the use of pain medication attenuates depressive symptoms and sleep disturbance. It should be noted that this finding does not deny the effect of medication use. However, our findings may contribute to a shift from the use of OTC analgesics to the application of self-management.

### Limitations

Our study has several limitations. First, this study found that psychological inflexibility was associated with depressive symptoms and sleep disturbance using a longitudinal design. However, this finding did not directly demonstrate a causal relationship, that is, that psychological inflexibility exacerbated depressive symptoms and sleep disturbance. Future research examining the effects of improved psychological inflexibility on depressive symptoms and sleep disturbance upon a follow-up of a patients undergoing treatment for chronic pain based on an ACT model will contribute to testing the possible causal relationships of psychological inflexibility with depressive symptoms and sleep disturbance among individuals with chronic pain.

In relation to this issue, this study measured depressive symptoms and sleep disturbance in women with chronic pain as an indicator of pain outcomes, rather than pain-related distress. Specifically, depressive symptoms and sleep disturbance measured in this study were also affected by other factors as well as chronic pain.

Second, our findings may not be generalizable to clinical populations with chronic pain because almost our entire sample comprised non-clinical women (87.5%). However, our findings may also be useful in clinical populations with chronic pain. This is because, even when limiting inpatient or regular outpatient treatments, psychological inflexibility was correlated with higher levels of depressive symptoms (*r* = 0.676, *p* < 0.001, *n* = 40) and sleep disturbance (*r* = 0.364, *p* = 0.021, *n* = 40). Additionally, approximately 33.4% of women with chronic pain in our sample reported severe pain intensity; the mean score of pain intensity (*M* = 5.53, SD = 1.83) in our sample was the same as or slightly higher than that in Asian clinical patients with chronic pain (*M* = 4.69, SD = 2.21) [24].

Third, although pain intensity was associated with higher levels of depressive symptoms, it could not predict the severity of sleep disturbance in women with chronic pain. Generally, existing studies [32,33] have shown that pain intensity is associated with higher levels of sleep disturbance in individuals with chronic pain; however, our results were inconsistent with those of previous studies. Our data showed that sleep disturbance was more severe in women with chronic pain than in women without such pain, suggesting that our data were valid. However, we cannot address this issue using only our data.

## 5. Conclusions

Regardless of these limitations, psychological inflexibility was longitudinally associated with higher levels of depressive symptoms and sleep disturbance in a Japanese sample of female college students with chronic pain, even when controlling for factors such as the effects of pain intensity, whether they consulted a doctor, and whether pain medication was used. This result confirms the effect of psychological inflexibility for individuals with chronic pain on negative pain outcomes in an Asian context. Therefore, knowing the findings of our study, improved self-management interventions aimed at reducing psychological inflexibility may be developed with the possible incorporation of ACT for individuals who are at higher risk of experiencing negative pain outcomes, such as those experiencing chronic pain. Aside from improving their associated symptoms, this may help lessen their need for OTC medication. Additionally, our findings can aid medical professionals and healthcare workers in providing optimal care for this target population and for other individuals experiencing chronic pain.

## Figures and Tables

**Table 1:** Medical treatment in the hospital, medication use, pain intensity, and site of pain in a sample of women with chronic pain (*n* = 320).

Medical Status		Our Sample	
		*n*	%
Medical treatment in the hospital			
	Inpatient treatment	6	1.9
	Regular outpatient treatment	34	10.6
	Formal medical advice	36	11.3
	No medical treatment	244	76.3
Medication use			
	Prescription medication	47	14.7
	Nonprescription mediation	117	36.6
	No medication	156	48.8
Pain intensity			
	Low (0–3)	43	13.4
	Moderate (4–6)	170	53.1
	Severe (7–10)	107	33.4
Pain site			
	Menstrual pain	114	35.6
	Head	98	30.6
	Abdomen (non-menstrual pain)	61	19.1
	Low back	21	6.6
	Shoulder or neck	9	2.8
	Other sites	17	5.3

**Table 2:** Means and standard deviations of psychological inflexibility, depressive symptoms, and sleep disturbance scores in our study and in comparative samples.

Variable	Women with Chronic Pain		Women without Chronic Pain		*t* Value	*p* Value	Comparative Sample	
	(*n* = 320)		(*n* = 90)				(*n* = 473)	
	Mean	SD	Mean	SD			Mean	SD
Psychological inflexibility	18.31	10.15	16.27	8.36	1.75	0.081	18.73	9.35
Depressive symptoms	7.44	5.72	6.07	4.85	2.08	0.038	6.81	4.73
Sleep disturbance	12.09	4.13	10.66	4.27	2.88	0.004		

Note. The comparative sample is Japanese female college students with chronic pain [18]. Potential ranges are 0 to 42 for psychological inflexibility, 0 to 27 for depressive symptoms, and 0 to 24 for daytime sleepiness.

**Table 3:** Hierarchical multiple regression analysis on depressive symptoms score with medical treatment, medication use, pain intensity, and psychological inflexibility scores as an explanatory variable.

Explanatory Variable	B	95% CI		*t* Value	*p* Value
		LL	UL		
Step 1					
Medical treatment	1.41	−0.05	2.87	1.91	0.057
Medication use	−1.54	−2.87	−0.21	−2.28	0.024
Pain intensity	1.54	0.52	2.55	2.98	0.003
*R*, *R*^2^, *p* value			0.21	0.04	0.003
Step 2					
Medical treatment	1.25	0.05	2.46	2.04	0.042
Medication use	−0.49	−1.61	0.63	−0.86	0.388
Pain intensity	1.04	0.20	1.89	2.43	0.016
Psychological inflexibility	0.31	0.26	0.37	12.08	<0.001
*R*, *R*^2^, *p* value			0.59	0.35	<0.001

Note. CI is Confidence Interval, LL and UL are lower and upper limits, respectively.

**Table 4:** Hierarchical multiple regression analysis on daytime sleepiness score with medical treatment, medication use, pain intensity, and psychological inflexibility scores as an explanatory variable.

Explanatory Variable	B	95% CI		*t* Value	*p* Value
		LL	UL		
Step 1					
Medical treatment	−0.37	−1.44	0.70	−0.68	0.498
Medication use	0.29	−0.69	1.27	0.59	0.556
Pain intensity	0.47	−0.27	1.22	1.25	0.212
*R*, *R*^2^, *p* value			0.10	0.01	0.374
Step 2					
Medical treatment	−0.41	−1.46	0.65	−0.76	0.450
Medication use	0.53	−0.44	1.51	1.07	0.284
Pain intensity	0.36	−0.38	1.10	0.96	0.337
Psychological inflexibility	0.07	0.03	0.12	3.15	0.002
*R*, *R*^2^, *p* value			0.20	0.04	0.012

Note. CI is Confidence Interval, LL and UL are lower and upper limits, respectively.
